# Supplementary material for: Sex differences in physical performance by age, educational level, ethnic groups and birth cohort: The Longitudinal Aging Study Amsterdam
Source: PLoS One. 2019 Dec 18;14(12):e0226342. doi: 10.1371/journal.pone.0226342 (PMC6919600; doi:10.1371/journal.pone.0226342)
Supplement: S1 Table — Percentage and number of participants for each physical performance measurement (percentage of total at same follow-up measurement). (DOCX) [file pone.0226342.s001.docx]

|  |  | **Baseline** | **FU #1** | **FU #2** | **FU #3** | **FU #4** | **FU #5** | **FU #6** | **FU #7** |
| --- | --- | --- | --- | --- | --- | --- | --- | --- | --- |
| **Birth cohort 1927-1937** |  |  |  |  |  |  |  |  |  |
| Gait speed | **Men** | 447 [95.7] | 381 [89.6] | 355 [92.9] | 317 [90.3] | 258 [88.4] | 220 [88.0] | 175 [85.0] | 120 [75.5] |
|  | **Women** | 486 [97.4] | 426 [92.2] | 373 [87.6] | 336 [84.2] | 294 [80.8] | 263 [81.2] | 222 [76.8] | 158 [70.5] |
| Chair stand | **Men** | 424 [90.8] | 369 [86.8] | 344 [90.1] | 301 [85.7] | 246 [84.2] | 209 [83.6] | 162 [78.6] | 103 [64.8] |
|  | **Women** | 472 [94.6] | 406 [87.9] | 355 [83.3] | 318 [79.7] | 268 [73.6] | 236 [72.8] | 195 [67.5] | 140 [62.5] |
| Handgrip strength^a^ | **Men** | - | 116 [27.3] | 175 [45.8] | 302 [86.0] | 256 [87.7] | 209 [83.6] | 164 [79.6] | 113 [71.1] |
|  | **Women** | - | 121 [26.2] | 209 [49.1] | 327 [82.0] | 298 [81.7] | 260 [80.3] | 210 [72.7] | 159 [71.0] |
| Balance | **Men** | - | 385 [90.5] | 358 [93.7] | 317 [90.3] | 259 [88.7] | 222 [88.8] | 176 [85.4] | 118 [74.2] |
|  | **Women** | - | 427 [92.4] | 379 [89.0] | 342 [85.7] | 293 [80.5] | 266 [82.1] | 222 [76.8] | 157 [70.1] |
| **Birth cohort 1937-1947** |  |  |  |  |  |  |  |  |  |
| Gait speed | **Men** | 463 [97.5] | 399 [93.9] | 351 [89.3] | 314 [88.7] | 279 [90.3] | - | - | - |
|  | **Women** | 507 [96.2] | 430 [89.0] | 373 [84.8] | 339 [83.7] | 305 [84.3] | - | - | - |
| Chair stand | **Men** | 449 [94.5] | 393 [92.5] | 342 [87.0] | 305 [86.2] | 277 [89.6] | - | - | - |
|  | **Women** | 482 [91.5] | 410 [84.9] | 352 [80.0] | 330 [81.5] | 287 [79.3] | - | - | - |
| Handgrip strength | **Men** | 433 [91.2] | 390 [91.8] | 345 [87.8] | 311 [87.9] | 280 [90.6] | - | - | - |
|  | **Women** | 483 [91.7] | 429 [88.8] | 377 [85.7] | 337 [83.2] | 298 [82.3] | - | - | - |
| Balance | **Men** | - | 402 [94.6] | 352 [89.6] | 314 [88.7] | 277 [89.6] | - | - | - |
|  | **Women** | - | 437 [90.5] | 376 [85.5] | 345 [85.2] | 306 [84.5] | - | - | - |

**Supplementary Table 1. Number participants per follow-up (FU) measurement for longitudinal birth cohorts 1927-1937 and 1937-1947.**

Missing values in physical performance due to refusing, being unable to perform the test or because no measurements were performed (only short interview). Number of participants included in the analysis [percentage of total participants at the corresponding measurement]. Baseline measurements of balance were not conducted in both birth cohorts and of handgrip strength not in birth cohort 1927-1937.

^a^Only measured in persons aged 60 years and older
