# Supplementary material for: Sex differences in physical performance by age, educational level, ethnic groups and birth cohort: The Longitudinal Aging Study Amsterdam
Source: PLoS One. 2019 Dec 18;14(12):e0226342. doi: 10.1371/journal.pone.0226342 (PMC6919600; doi:10.1371/journal.pone.0226342)
Supplement: S2 Table — (DOCX) [file pone.0226342.s002.docx]

**Supplementary Table 2. Number participants for each physical performance measurement (percentage of total) for cross-sectional birth cohorts 1947-1957 and migration cohort.**

|  | **Birth cohort 1947-1957** | | **Migration cohort 1948-1958** | |
| --- | --- | --- | --- | --- |
| *Physical performance* | **Men** | **Women** | **Men** | **Women** |
| Gait speed | 489 [98.6] | 519 [98.5] | 264 [96.0] | 182 [89.7] |
| Chair stand | 474 [95.6] | 490 [93.0] | 210 [76.4] | 142 [70.0] |
| Handgrip strength | 430 [86.7] | 437 [82.9] | 172 [62.5] | 142 [70.0] |
| Balance | 493 [99.4] | 523 [99.2] | 270 [98.2] | 188 [92.6] |

Missing values in physical performance due to refusing, being unable to perform the test or because no measurements were performed (only short interview). Number of participants included in the analysis [percentage of total participants at the corresponding measurement].
