# Supplementary material for: Sex differences in physical performance by age, educational level, ethnic groups and birth cohort: The Longitudinal Aging Study Amsterdam
Source: PLoS One. 2019 Dec 18;14(12):e0226342. doi: 10.1371/journal.pone.0226342 (PMC6919600; doi:10.1371/journal.pone.0226342)
Supplement: S3 Table — (DOCX) [file pone.0226342.s003.docx]

**Supplementary Table 3. Effect modification of sex differences in physical aging by education, ethnic groups and birth cohort.**

|  |  | **Gait speed** | **Chair stand** | **Handgrip**  **strength** | **Balance^d^** |
| --- | --- | --- | --- | --- | --- |
| **By education^a^** |  |  |  |  |  |
| Sex (female) * Education  (high vs low) | Birth cohort 1927-37 | p=0.657 | p=0.242 | p=0.474 | p=0.608 |
|  | Birth cohort 1937-47 | p=0.437 | **p=0.046** | p=0.624 | p=0.180 |
| Sex (female) * Education  (middle vs low) | Birth cohort 1927-37 | p=0.657 | p=0.780 | p=0.124 | p=0.357 |
|  | Birth cohort 1937-47 | p=0.237 | **p=0.001** | p=0.946 | p=0.923 |
| Sex (female) * Education  (high vs middle) | Birth cohort 1927-37 | p=0.477 | p=0.122 | p=0.390 | p=0.676 |
|  | Birth cohort 1937-47 | **p=0.017** | p=0.118 | p=0.613 | p=0.174 |
| Sex * Age (y) * Education  (high vs low) | Birth cohort 1927-37 | p=0.617 | p=0.283 | p=0.251 | p=0.577 |
|  | Birth cohort 1937-47 | p=0.095 | p=0.458 | p=0.610 | p=0.412 |
| Sex * Age (y) * Education  (middle vs low) | Birth cohort 1927-37 | p=0.142 | p=0.865 | p=0.806 | p=0.451 |
|  | Birth cohort 1937-47 | p=0.357 | p=0.614 | p=0.514 | p=0.206 |
| Sex * Age (y) * Education  (high vs middle) | Birth cohort 1927-37 | p=0.270 | p=0.301 | p=0.314 | p=0.839 |
|  | Birth cohort 1937-47 | p=0.360 | p=0.773 | p=0.937 | p=0.626 |
| Sex * Age * Age (y^2^) * Education  (high vs low) | Birth cohort 1927-37 | p=0.667 | p=0.538 | p=0.399 | p=0.872 |
|  | Birth cohort 1937-47 | p=0.524 | p=0.289 | p=0.468 | p=0.519 |
| Sex * Age * Age (y^2^) * Education  (middle vs low) | Birth cohort 1927-37 | p=0.555 | **p=0.027** | p=0.885 | p=0.250 |
|  | Birth cohort 1937-47 | p=0.667 | p=0.614 | p=0.990 | p=0.293 |
| Sex *Age * Age (y^2^) * Education  (high vs middle) | Birth cohort 1927-37 | p=0.657 | p=0.077 | p=0.280 | p=0.262 |
|  | Birth cohort 1937-47 | p=0.524 | p=0.488 | p=0.926 | p=0.636 |
| **By ethnic group^b^** |  |  |  |  |  |
| Sex (female) * Ethnicity (Dutch) |  | p=0.222 | **p<0.001** | **p<0.001** | p=0.902 |
| **By birth cohort^c^** |  |  |  |  |  |
| Sex (female) * Birth cohort (1937-47) |  | p=0.415 | p=0.289 | **p=0.026** | p=0.308 |
| Sex * Age (y) * Birth cohort (1937-47) |  | p=0.537 | p=0.243 | **p=0.037** | p=0.875 |
| Sex * Age * Age (y^2^) * Birth cohort (1937-47) |  | p=0.464 | p=0.656 | p=0.786 | p=0.065 |

Beta [95% confidence interval], p-value

^a^Longitudinal data of LASA birth cohort 1927-1937 (1) and of LASA birth cohort 1937-1947 (2)

^b^Cross-sectional data of LAS birth cohort 1947-1957 and Migration cohort 1948-1958

^c^Longitudinal data of LASA birth cohort 1937-1947 versus 1927-1937

^d^OR [95% confidence interval], p-value
